# Precipitous Declines in Northern Gulf of Mexico Invasive Lionfish Populations Following the Emergence of an Ulcerative Skin Disease

**DOI:** 10.1038/s41598-020-58886-8

**Published:** 2020-02-04

**Authors:** Holden E. Harris, Alexander Q. Fogg, Micheal S. Allen, Robert N. M. Ahrens, William F. Patterson

**Affiliations:** 1 School of Natural Resources and Environment, Institute of Food and Agriculture Sciences, University of Florida, PO Box 116455, Bldg. 0724, 103 Black Hall, Gainesville, FL 32611 USA; 2 Fisheries and Aquatic Sciences, School of Forest Resources and Conservation, Institute of Food and Agriculture Sciences, University of Florida, 7922 NW 71st Street, Gainesville, FL 32653 USA; 3 Okaloosa County Board of County Commissioners, Destin – Fort Walton Beach, FL 32548 USA; 4 Nature Coast Biological Station, Institute of Food and Agriculture Sciences, University of Florida, PO Box 878, 552 1st Street, Cedar Key, FL 32625 USA

**Keywords:** Ecology, Ecology, Community ecology, Ecological epidemiology, Ecological modelling, Evolutionary ecology, Invasive species, Population dynamics

## Abstract

Invasive Indo-Pacific lionfish *Pterois volitans/miles* have become well-established in many western Atlantic marine habitats and regions. However, high densities and low genetic diversity could make their populations susceptible to disease. We examined changes in northern Gulf of Mexico (nGOM) lionfish populations following the emergence of an ulcerative skin disease in August 2017, when estimated disease prevalence was as high as 40%. Ulcerated female lionfish had 9% lower relative condition compared to non-ulcerated females. Changes in lionfish size composition indicated a potential recruitment failure in early summer 2018, when the proportion of new recruits declined by >80%. Remotely operated vehicle surveys during 2016–2018 indicated lionfish population density declined in 2018 by 75% on natural reefs. The strongest declines (77–79%) in lionfish density were on high-density (>25 lionfish per 100 m^2^) artificial reefs, which declined to similar levels as low-density (<15 lionfish per 100 m^2^) artificial reefs that had prior lionfish removals. Fisheries-dependent sampling indicated lionfish commercial spearfishing landings, commercial catch per unit effort (CPUE), and lionfish tournament CPUE also declined approximately 50% in 2018. Collectively, these results provide correlative evidence for density-dependent epizootic population control, have implications for managing lionfish and impacted native species, and improve our understanding of biological invasions.

## Introduction

Biological invasions have caused profound ecological and economic damages in myriad ecosystems around the globe^1^. However, the mechanisms that determine whether introduced species become invasive are often poorly understood^2,3^ and those that do establish invasive populations often undergo unforeseen crashes and recoveries^1,4^. While population booms may be partially explained by life history attributes^1^, the causes of crashes are generally unknown^4,5^. Crashes my indirectly result from founder effects, including genetic bottlenecks and inbreeding^6^, that can increase susceptibility to genetic drift^7^ or disease^8^. Forecasting invasions is further confounded by contemporary interactions of ecological and evolutionary processes^9^ as invasive species exert selective pressure on native communities, which in turn influence invasive species’ population dynamics. Ultimately, invasion success for many species may be more dependent on their ability to evolve within their invasive range than on inherent physiological attributes^10^.

Indo-Pacific lionfish (*Pterois volitans/miles* complex, hereafter ‘lionfish’) in the western Atlantic Ocean have reached the final stage of a biological invasion, i.e., being widespread and dominant in many habitats with individuals dispersing, surviving, and reproducing at multiple sites across a broad geographic range^2^. A generalist diet^11^, novel morphological traits^12^, broad physiological tolerances to temperature^13^ and salinity^14^, and opportunistic life history characteristics^15–17^ have facilitated their establishment in a wide diversity of marine habitats, including tropical coral continuous and patch reefs, artificial reefs (ARs), tropical and subtropical estuaries, seagrass beds, mangroves, mesophotic reefs, and the upper continental slope^18–21^. Their invasive range has expanded through the North Atlantic Basin, Caribbean Sea, Gulf of Mexico, and Guyana Basin^21^, with densities reported up to *c*. 0.3 lionfish per 100 m^2^ on tropical and subtropical natural reefs (NRs)^18,19^ and >30 lionfish per 100 m^2^ on ARs^19^. The invasion’s impacts are considered massive^2^ due to region-wide deleterious effects on reef fish communities^22–24^ and ecosystem processes^25–27^.

Lionfish densities in their native Indo-Pacific range are substantially lower than those reported among various systems in the western Atlantic^28,29^, but the principal mechanisms that control native populations remain largely unknown^20,21^. The lionfish invasion history in The Bahamas includes a rapid increase of population densities during 2005–2009^18^, a peak in 2010–2011^30^, and then a levelling off or decrease in some areas between 2012 and 2015^30^. Their northern Gulf of Mexico (nGOM) invasion similarly included a rapid population increase during 2010–2014 before leveling off in 2015–2017^19^. These invasive populations do not appear to be controlled by native predators^20,31–34^ (but see Mumby *et al*. 2011) and shown resistance to parasites in their invaded range^35–39^. Recent studies indicate the population stagnations observed in The Bahamas and nGOM may be a result of intraspecific competition^40,41^ and local resource depletion^22,24,42^. Higher lionfish density correlates to increased foraging distances^41^ and higher rates of cannibalism^43^, resulting in individuals that have a smaller size-at-age and lower relative condition^19,44^.

High population densities^18,19^ and low genetic diversity in the western Atlantic^45–47^ suggest invasive lionfish could also be vulnerable to pathogenic control^8,48^. In August 2017, the first reported disease was documented in lionfish collected from nGOM high-density (>25 lionfish per 100 m^2^) nGOM ARs^49^. Ulcerated lionfish were subsequently reported from the northeast and southern Gulf of Mexico, the Caribbean, and tropical west Atlantic^49^. Preliminary pathological investigations have, so far, not identified an infectious cause or a possible emerging pathogen (pers. comm., T. Cody/R. Yanong, Florida Fish and Wildlife Research Institute/University of Florida). Better understanding of the disease etiology could inform its potential lethal and sublethal effects on lionfish and whether it could transmit to native fishes^50^.

**Figure 1:**
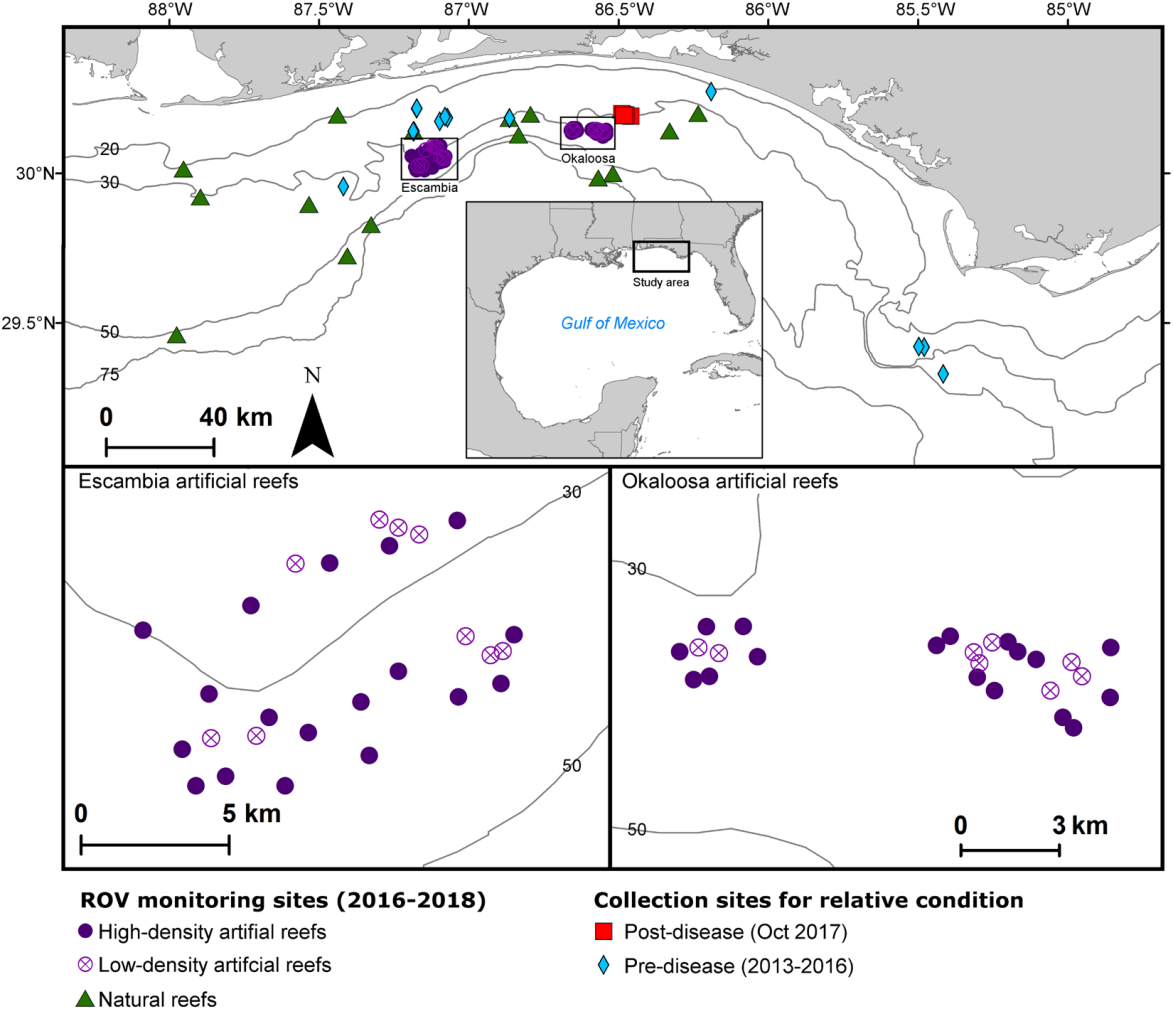
Study area and reef site locations monitoring northern Gulf of Mexico (nGOM) invasive lionfish populations and the lionfish ulcerative skin disease. Regional lionfish sampling in the study area (29–30° N, 85–88° W) enabled examination of lionfish size composition during 2014–2018 and disease prevalence following its initial report in August 2017. Fisheries catch data were evaluated in the study area from commercial spearfishing landings during 2015–2018 and lionfish tournaments during 2017–2019. Artificial reef (AR) and natural reef (NR) locations are indicated for collection sites of lionfish examined for relative condition pre- and post- disease emergence and population density monitoring. Population density monitoring was conducted with remote operated vehicle surveys during 2016–2018 on NRs throughout the study area and two groups of ARs offshore of Escambia and Okaloosa counties, Florida. High-density ARs were monitored only; low-density ARs had experimental removals in 2014 (Escambia^22^) or 2016 (Okaloosa^51^) prior to the appearance of ulcerated fish. Isobaths for depths of 20-, 30-, 50-, and 75-m are shown. Map created with ArcGIS Desktop 10.7.1.

We examined changes in nGOM lionfish populations following the appearance of lionfish ulcerative skin disease. A variety of data from the nGOM (Fig. 1) were used to examine: 1) disease prevalence during 2017–2019, 2) relative condition of ulcerated versus apparently healthy lionfish, 3) recruitment patterns inferred from population size composition of nGOM lionfish sampled during 2014–2018, 4) lionfish population densities assessed with remotely operated vehicle (ROV) video surveys on ARs and NRs during 2016–2018, and 5) fisheries catch per unit effort (CPUE) from commercial spearfishing landings during 2014–2018 and lionfish tournaments during 2017–2019. Results are presented and inferences drawn with respect to potential causes for the lionfish population declines, boom-bust population cycles, and pathogenic control of invasive species to consider their implications for lionfish management and invasion ecology.

## Results

### High ulcer prevalence was observed in August 2017

**Figure 2:**
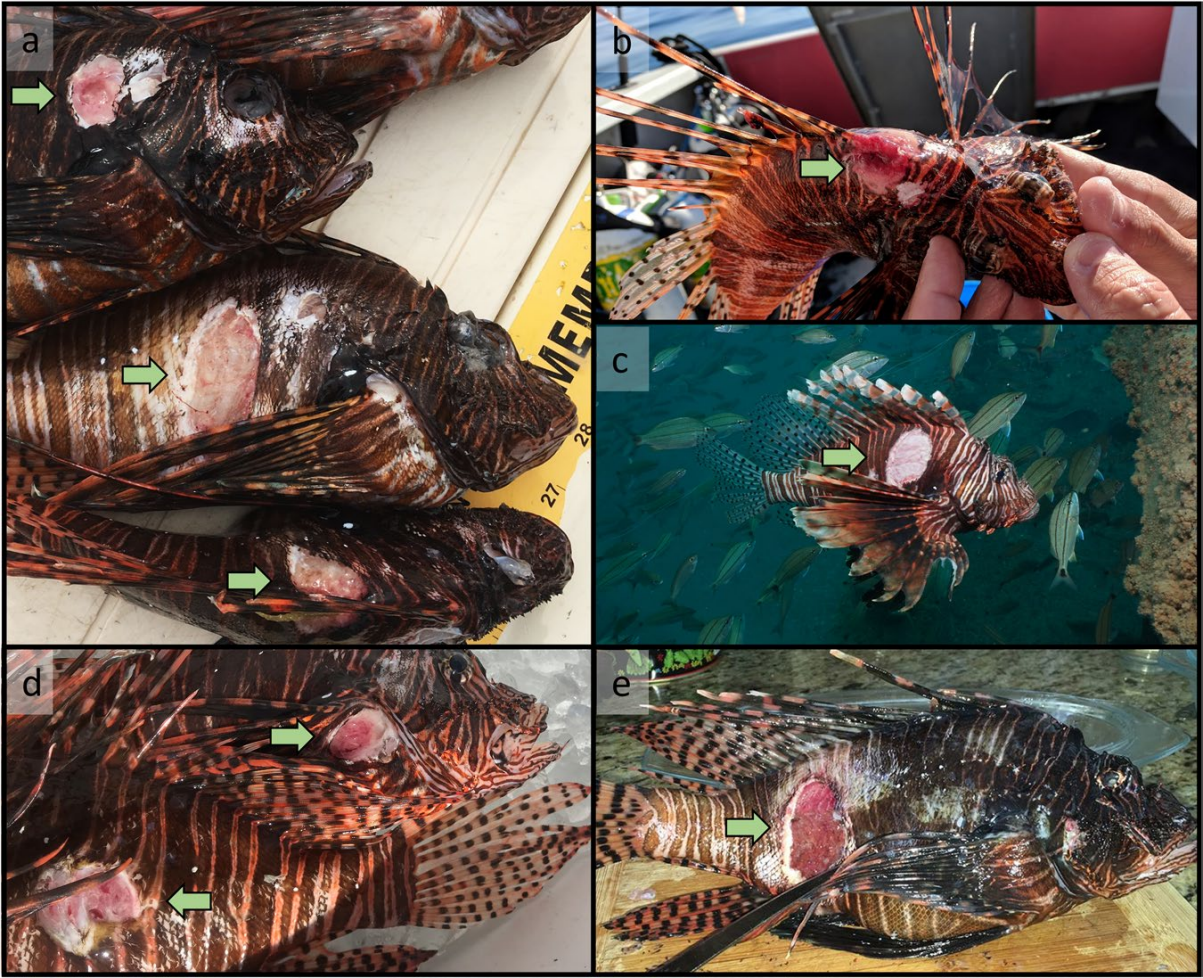
Images of lionfish presenting signs of an ulcerative skin disease (indicated with arrows). (**a**) Ulcerated lionfish 15 d following their initial report in the northern Gulf of Mexico in August 2017 (Apalachicola, FL, photo: A. Fogg). Conspicuous ulcers were observed in various anatomical locations, including (**b**) the dorsal fin spines base (October 2017, Destin, FL, photo: H. Harris) and (**c**) posterior the pectoral fin base (image taken underwater, December 2019, Destin, FL, photo: A. Fogg). Lionfish were also reported to the authors with similar signs elsewhere in the invaded range, including (**d**) Grand Cayman Island (September 2017, photo J. Washington) and (**e**) Bonaire (March 2018, photo: J. Spruit).

Lionfish with the ulcerative skin disease presented skin ulcers that varied in size and location (Fig. 2). Ulcers were easily identifiable and partial ulcer development was not observed. Deep ulcers through the epidermis exposed skeletal muscle with a sloughing of necrotic tissue. Following the first observation of the ulcerative skin disease on nGOM ARs on August 5, 2017, ulcer occurrence was documented from lionfish collected via commercial harvest during August, October, and December 2017. Ulcer occurrence was then documented during monthly lionfish size composition sampling conducted February 2018–June 2019.

**Figure 3:**
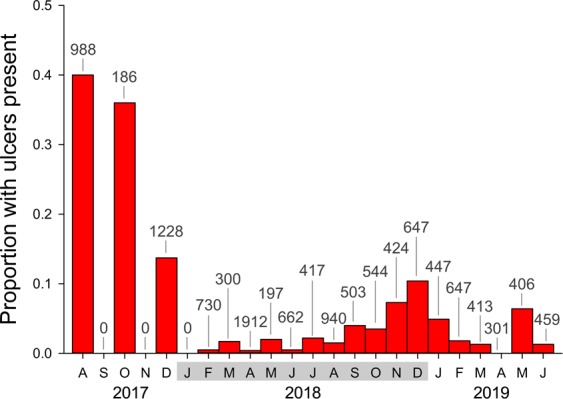
Ulcer prevalence (proportion of fish sampled with ulcers identified by visual examination) by month on northern Gulf of Mexico lionfish following the initial report of ulcerated fish in August 2017. Monthly lionfish sample size is indicated above each bar. No fish were sampled in September 2017, November 2017, or January 2018.

Ulcers were observed on 861 of 12,159 lionfish examined. Ulcer prevalence (i.e., the proportion of visually identified ulcerated lionfish within a population sample) was highest during the first month of its emergence in August 2017, with 395 of 988 lionfish (40%) presenting ulcers (Fig. 3). Prevalence subsequently declined that December to 14% (168 of 1228 lionfish) and further declined to <3% during Jan–Oct 2018. Prevalence then increased in Nov–Dec 2018 to 7–10% before declining again to 0–6% during Jan–Jun 2019.

### Relative condition was lower in ulcerated lionfish

Sex-specific lionfish relative condition factor (K_n_) was estimated for lionfish sampled in 2017 as the ratio of an individual’s measured weight to its sex-specific predicted weight-at-length ($\hat{{\rm{W}}}$). These $\hat{{\rm{W}}}$ allometric parameters were computed from 282 males and 316 females sampled from nGOM NRs prior to disease emergence during 2013–2016. ANOVA results indicated a significant difference between male and female scaling *b* values (F_(1, 594)_ = 9.14; P = 0.003). Thus, female $\hat{{\rm{W}}}$ values were calculated with *a* = 4.67e-10 and *b* = 3.62 and male $\hat{{\rm{W}}}$ with *a* = 1.66e-10 and *b* = 3.42. K_n_ was then evaluated for 338 lionfish sampled from nGOM NRs in October–November 2017, of which 39 lionfish (20 males and 19 females) presented active skin ulcers. Sampled lionfish size in total length (TL) ranged from 118–367 mm. Ulcerated lionfish TL ranged from 212–352 mm and showed no apparent trend in ulcer occurrence by size. A two-way analysis of variance (ANOVA) and Tukey honest significant differences (HSD) test was used to test the effect of ulcer presence, sex, and their interaction on differences in K_n._

**Figure 4:**
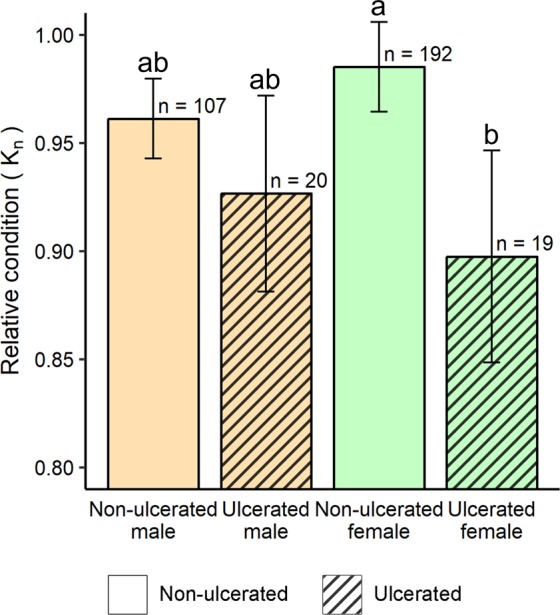
Sex-specific mean (±95% CI) relative condition (K_n_) for non-ulcerated and ulcerated lionfish with number of lionfish collected (n) indicated. Letters above bars indicate significantly different group means (Tukey HSD, P < 0.05). Lionfish were collected during October–November 2017 from northern Gulf of Mexico (nGOM) natural reefs (NRs). Sex-specific predicted weight-at-length models were developed from 598 lionfish sampled before the appearance of the ulcerative disease during 2013–2016 on similar nGOM NRs.

ANOVA results indicated K_n_ was significantly lower for ulcerated lionfish compared to non-ulcerated lionfish (F_(1, 334)_ = 7.81; P = 0.006). Mean K_n_ was 0.93 (95% CI = 0.88–0.97) in ulcerated males and 0.90 (CI = 0.84–0.95) in ulcerated females (Fig. 4, hatched bars). Multiple comparison tests indicated this significant decline was driven by the 8.8% lower K_n_ in ulcerated females (Tukey HSD, P = 0.025). No other pairwise comparisons were significant in the Tukey HSD test. Mean K_n_ for apparently healthy (non-ulcerated) lionfish collected in 2017 was 0.96 (CI = 0.94–0.98) in males and 0.99 (CI = 0.96–1.00) in females (Fig. 4, empty bars). ANOVA results indicated K_n_ was not significantly different based on sex (F_(1, 334)_ = 2.23; P = 0.140) nor the interaction between ulcer prevalence and sex (F_(1, 334)_ = 1.46; P = 0.230). It is notable that K_n_ in apparently healthy lionfish was 4% lower in males and 1% lower in females compared to what would be predicted by the sex-specific weight-at-length models. Potential explanations for this difference include seasonal changes in gonadosomatic index^17^ or density-dependent competition for higher density populations in 2013–2016^19^. Also, lionfish collections prior to the disease were from NR habitat like the 2017 collections, but from a broader area (Fig. 1).

### Size composition changed and the proportion of new recruits declined in 2018

**Figure 5:**
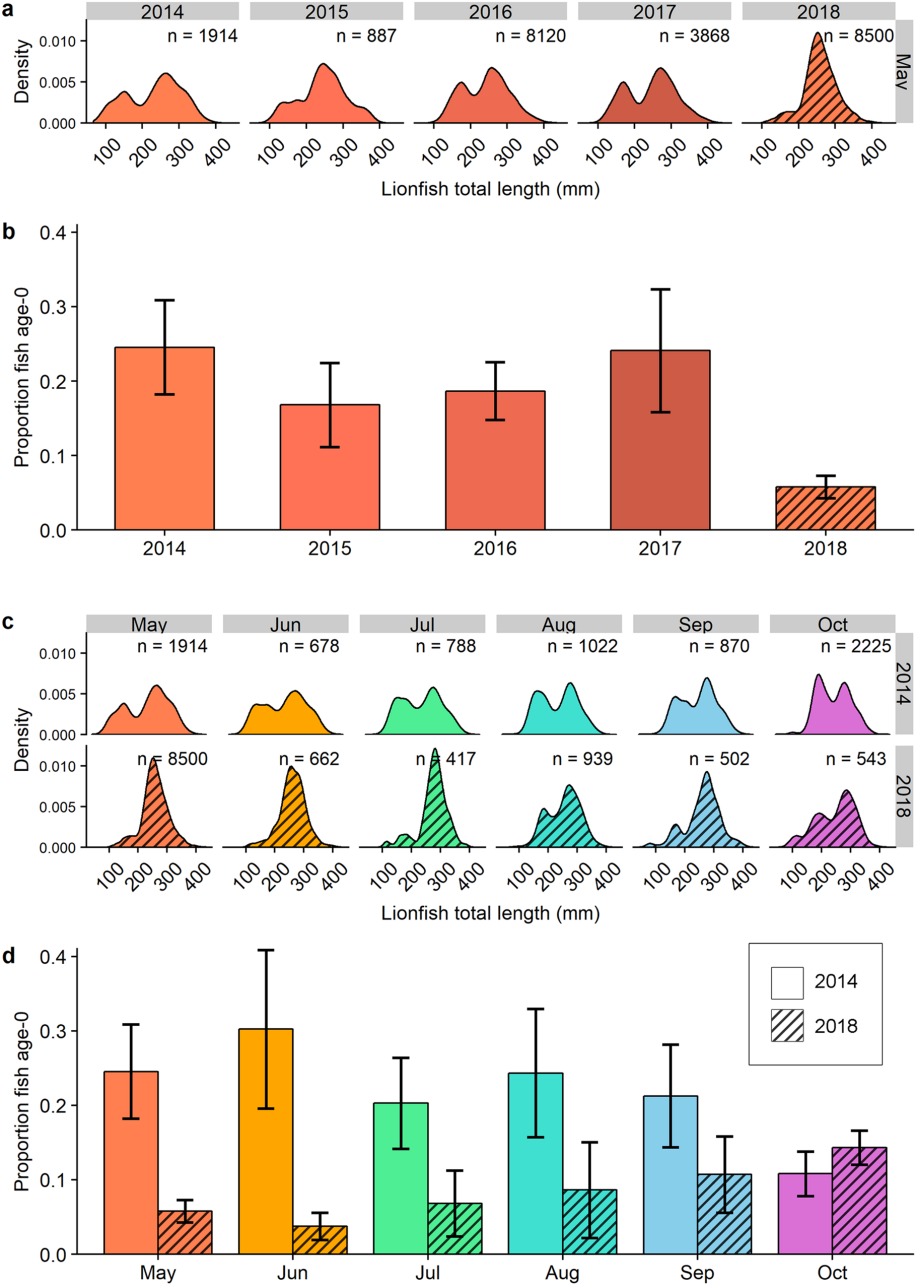
Northern Gulf of Mexico lionfish population size composition and recruitment during 2014–2018. (**a**) Density plots of lionfish total length (TL) sampled during May 2014–2018 with number of lionfish sampled per month (n) indicated. (**b**) Mean (±95% CI) proportion of age-0 newly recruited lionfish among samples per sampling event ($\hat{{\rm{P}}}$) during May 2014–2018. (**c**) Density plots of lionfish TL sampled during May–October 2014 (solid plot) and May–October 2018 (hatched plot). (**d**) Paired means (±95% CI) of $\hat{{\rm{P}}}$ sampled May–October in 2014 (solid bars) and 2018 (hatched bars).

Northern GOM lionfish size composition was assessed for 31,073 lionfish harvested via spearfishing from 201 sampling events (collection days) during 2014–2018. Length frequency density plots of lionfish collected during 2014–2017 show bimodal distributions with local minima *c*. 175–200 mm, similar to the estimated demarcation between newly recruited (age-0) and older lionfish (Fig. 5A,C, solid plots). In contrast, density plots for lionfish collected in early summer 2018 show a relatively unimodal distribution (Fig. 5A,C, hatched plots). Changes in size composition and recruitment were examined by differences in the mean proportion of age-0 lionfish ($\hat{{\rm{P}}}$) among monthly population samples. Differences in $\hat{{\rm{P}}}$ were evaluated with a nested binomial generalized linear model (GLM) with sampling events as the experimental unit to test differences 1) between months within the same year and 2) between years within the same month (Table 1).

**Table 1:** Proportion of newly recruited age-0 lionfish ($\hat{{\rm{P}}}$) among northern Gulf of Mexico lionfish population samples during 2014–2018. Differences in $\hat{{\rm{P}}}$ were tested with a binomial generalized linear model (GLM) for month nested within year. Experimental units were sampling events weighted by logged number of lionfish sampled per month. GLM outputs show the logit-linked parameter estimates for $\hat{{\rm{P}}}$, 95% confidence intervals (CI) around $\hat{{\rm{P}}}$, z-score (z), and p-values (P). Hypothesis testing (z- and p-values) for differences in $\hat{{\rm{P}}}$ prior to disease emergence (May–Oct 2014 and May 2015–2017) are relative to the intercept (May 2014). Hypotheses testing of $\hat{{\rm{P}}}$ during May–Oct 2018 are relative to the same sampling month in 2014.

Sampling date		Sampling events (#)	Lionfish sampled	Proportion Age-0 ($\hat{{\rm{P}}}$)	95% CI	z	P
2014	May *(Intercept)*	19	1,914	0.39	0.24–0.63	−3.85	**<0.001**
2014	Jun	7	678	0.34	0.13–0.87	−0.30	0.765
2014	Jul	9	788	0.33	0.14–0.80	−0.36	0.722
2014	Aug	9	1,022	0.30	0.12–0.73	−0.56	0.574
2014	Sep	9	870	0.21	0.08–0.56	−1.23	0.219
2014	Oct	11	2,225	0.12	0.05–0.32	−2.39	**0.017**
2015	May	7	887	0.21	0.08–0.60	−1.14	0.255
2016	May	18	8,120	0.21	0.10–0.43	−1.73	0.084
2017	May	14	3,868	0.26	0.12–0.55	−1.07	0.287
2018	May	20	8,500	0.06	0.02–0.16	−3.83	**<0.001**
2018	Jun	5	662	0.05	0.01–0.46	−1.81	0.070
2018	Jul	2	417	0.07	0.01–1.06	−1.19	0.235
2018	Aug	3	939	0.09	0.02–0.76	−1.26	0.207
2018	Sep	2	502	0.12	0.03–1.76	−0.54	0.590
2018	Oct	5	543	0.17	0.13–2.34	0.48	0.629

GLM results indicated $\hat{{\rm{P}}}$ during May 2014–2017 was 0.17–0.22 (Fig. 5B) and 0.23–0.28 during May–Oct 2017 (Fig. 5D empty bars). In 2014, $\hat{{\rm{P}}}$ decreased throughout the summer to be significantly different (P < 0.017) in October compared to May that same year, presumably as the 2014 age-0 cohort grew larger and surpassed the cutoff size. In contrast to 2014–2017, $\hat{{\rm{P}}}$ during May–Aug 2018 declined to 0.05–0.09 (Fig. 5D). GLM results indicate $\hat{{\rm{P}}}$ was 0.06 in May 2018 and 84% lower compared to May 2014 (Table 1, P < 0.001). Model results also indicated mean $\hat{{\rm{P}}}$ was 0.05–0.09 during June–August 2018 and 70–86% lower than corresponding months in 2014 (Fig. 5D hatched vs. empty bars). These differences were not statistically significant in the GLM due to the low sample size using sampling events (n = 5, 2, and 3 respectively) per month (Table 1). However, the size of the effect along with number of lionfish sampled (n = 662, 417, and 923) suggest the difference may be biologically important. Mean $\hat{{\rm{P}}}$ increased throughout 2018, indicating a potential return of new recruits later that year. By October, $\hat{{\rm{P}}}$ was 0.17 and similar to what it was in October 2014 when $\hat{{\rm{P}}}$ was 0.12 (P = 0.629).

### Lionfish population density declines observed by ROV sampling

Lionfish population densities were estimated during 2016–2018 via ROV surveys (n = 338 sampling events) at 18 Okaloosa ARs, 27 Escambia ARs, and 15 nGOM NRs (Fig. 1). ARs included low-density (<15 lionfish per 100 m^2^) reefs that had experimental lionfish removals conducted in 2014 (Escambia)^22^ and 2016 (Okaloosa)^51^. High-density (>25 lionfish per 100 m^2^) ARs were control reefs during those experiments with no lionfish removed. Changes in lionfish densities over time (by sampling date) were evaluated with generalized linear mixed models (GLMMs) with repeated sites incorporated as a random effect. GLMMs were computed to test for differences in population densities within and between density treatments (i.e., low- versus high-density) over time for Okaloosa and Escambia ARs and over time for nGOM NRs (Table 2).

**Table 2:** Lionfish population density (fish per 100 m^2^) on Okaloosa artificial reefs (ARs), Escambia ARs, and northern Gulf of Mexico (nGOM) natural reefs (NRs) during 2016–2018 estimated via remotely operated vehicle surveys. Changes in mean lionfish population density over time were assessed with generalized linear mixed models (GLMMs) with site incorporated as a random effect. Okaloosa AR and Escambia AR GLMMs were fit with a negative binomial error distribution and the nGOM NR model was fit with a Poisson distribution. GLMM outputs show log-linked parameter estimates for mean lionfish population density, 95% confidence intervals (CI) around mean lionfish density, z-score (z), and p-values (P). Hypothesis testing (z- and p-values) were conducted for each model (Okaloosa ARs, Escambia ARs, and nGOM NRs) to evaluate differences in population density on high-density sites (>25 lionfish per 100 m^2^ and with no prior lionfish removals) relative to the intercept (initial sampling date) and low-density sites (<15 lionfish per 100 m^2^ with prior lionfish removals) relative to high-density sites within the same area and sampling date.

Lionfish density model		Sampling date	Density group	Reef sties	Mean density (100 m^−2^)	95% CI	z	P
Okaloosa ARs	2016	Oct *(Intercept)*	High	10	27.22	17.69–41.88	15.03	**<0.001**
	2016	Dec	High	10	23.41	16.88–32.94	−0.87	0.383
	2016	Feb	High	10	24.23	17.42–34.03	−0.67	0.500
	2017	Apr	High	10	25.86	18.51–36.20	−0.28	0.779
	2017	May	High	10	24.77	17.69–34.57	−0.56	0.575
	2017	Oct	High	10	21.78	15.52–30.76	−1.26	0.209
	2018	Mar	High	10	13.88	09.80–19.87	−3.78	**<0.001**
	2018	Jun	High	10	7.35	05.17–10.34	−7.17	**<0.001**
	2018	Oct	High	8	5.72	03.81–08.71	−7.61	**<0.001**
	2016	Dec	Low	8	5.17	02.72–10.34	−4.80	**<0.001**
	2017	Feb	Low	8	3.98	02.11–07.96	−5.07	**<0.001**
	2017	Apr	Low	8	6.30	03.15–12.11	−4.02	**<0.001**
	2017	May	Low	8	7.50	03.88–14.74	−3.63	**<0.001**
	2017	Oct	Low	8	15.61	08.17–29.97	−1.40	0.161
	2018	Mar	Low	8	18.29	09.36–35.28	−0.53	0.599
	2018	Jun	Low	8	11.94	06.11–23.60	−0.42	0.671
	2018	Oct	Low	8	10.44	05.22–20.87	0.99	0.324
Escambia ARs	2016	June *(Intercept)*	High	18	18.65	12.41–28.04	14.06	**<0.001**
	2017	Nov	High	9	31.71	17.90–56.32	1.81	0.070
	2018	May	High	18	8.39	05.22–13.43	−3.32	**0.001**
	2018	Aug	High	18	6.34	03.92–10.26	−4.42	**<0.001**
	2018	Dec	High	18	7.27	04.48–11.56	−3.92	**<0.001**
	2016	Jun	Low	9	8.02	03.92–16.41	−2.30	**0.022**
	2017	Nov	Low	9	8.56	03.80–19.34	−3.16	**0.002**
	2018	May	Low	9	5.87	02.85–12.34	−0.94	0.348
	2018	Aug	Low	9	3.04	01.46–06.53	−1.89	0.058
	2018	Dec	Low	9	4.51	02.18–09.46	−1.26	0.208
nGOM NRs	2016	Jul *(Intercept)*	High	15	0.24	0.15–0.38	−6.06	**<0.001**
	2017	Oct	High	15	0.35	0.24–0.52	1.98	**0.048**
	2018	May	High	7	0.29	0.15–0.57	0.54	0.592
	2018	Aug	High	15	0.20	0.13–0.30	−0.88	0.380
	2018	Oct	High	8	0.07	0.03–0.15	−3.11	**0.002**

**Figure 6:**
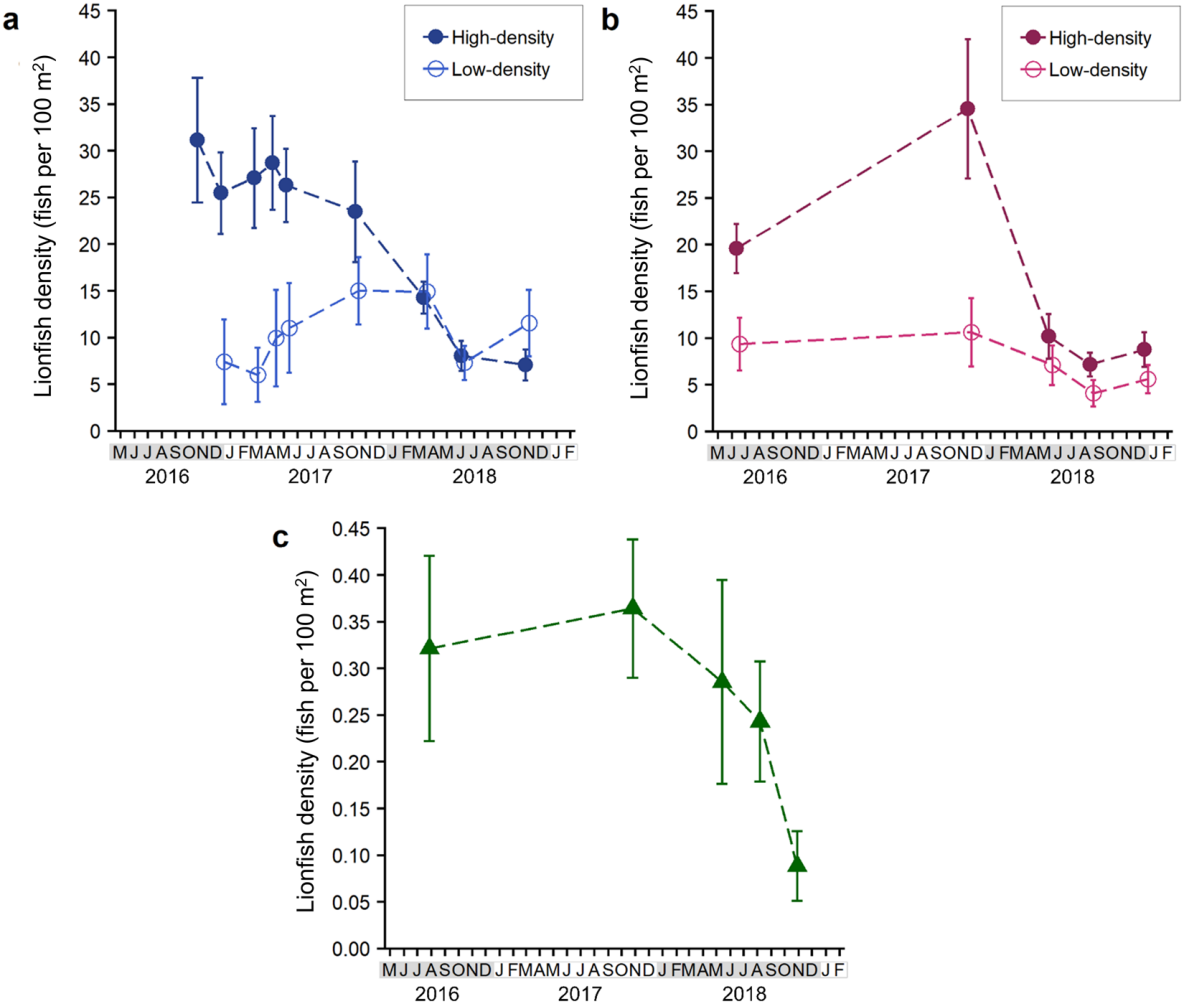
Mean (±SE) lionfish population density during May 2016 to December 2018 estimated via remotely operated vehicle (ROV) surveys. ROV surveys monitored (**a**) 8 low-density and 10 high-density Okaloosa artificial reefs (ARs), (**b**) 9 low-density and 18 high-density Escambia ARs, and c) 15 northern Gulf of Mexico natural reefs (NRs). Low-density ARs (initial density <15 fish per 100 m^2^) had experimental removals conducted in 2014 (Escambia^22^) and 2017 (Okaloosa^51^) prior to the appearance of ulcerated fish; high-density ARs (initial density > 25 fish per 100 m^2^) were control sites without removals. NR population densities (**c**) were ~100X lower than those on ARs, shown here with a different y-axis scale.

During 2016 and early 2017, mean lionfish population density on high-density ARs was 21–27 lionfish per 100 m^2^ on Okaloosa ARs and 19–31 lionfish per 100 m^2^ on Escambia ARs (Fig. 6A,B). Lionfish density on high-density ARs were significantly higher (*c*. 2–5X) than mean densities on low-density ARs in the same area (Table 2). Results from the GLMM analyses show lionfish density declines began in late 2017 and continued through 2018. Mean lionfish density on high-density Okaloosa ARs declined 79% between October 2016 (27 lionfish per 100 m^2^) and October 2018 (6 lionfish per 100 m^2^). By October 2017, mean lionfish density was no longer significantly different between initially high- and low-density Okaloosa ARs (Table 2). In fact, by October 2018 lionfish density on Okaloosa high-density ARs (6 lionfish per 100 m^2^) was less than the lionfish density on corresponding low-density ARs (10 lionfish per 100 m^2^), although the difference was not significant in the GLMM (P = 0.324). Similarly, lionfish densities at initially high-density Escambia ARs declined 77% from November 2017 (31 lionfish per 100 m^2^) to December 2018 (7 lionfish per 100 m^2^), by which time they were not significantly different from low-density ARs (5 lionfish per 100 m^2^, P = 0.208). Concurrent declines were also observed on low-density ARs, but were smaller in magnitude and proportion. Mean lionfish density on low-density Okaloosa ARs, which had relatively recent removals in October 2016, increased in density during early 2017 from 4 lionfish per 100 m^2^ in February up to 15 lionfish per 100 m^2^ in October 2017, then declined 23% by October 2018 (11 lionfish per 100 m^2^). Mean density on low-density Escambia ARs (lionfish removed in 2014) declined between June 2016 (9 lionfish per 100 m^2^) and December 2018 (6 fish per 100 m^2^). Lionfish densities on NRs were approximately two-orders of magnitude lower than ARs, similar to what has been reported in other studies in the region^19,51,52^. Mean lionfish density on NRs (Fig. 6C) increased significantly between 2016–2017 (P = 0.048, Table 2). Similar in timing and proportion to the declines on high-density ARs, densities then declined 75% between October 2017 (0.35 lionfish per 100 m^2^) and October 2018 (0.07 lionfish per 100 m^2^, P = 0.002).

### Regional declines observed in lionfish fisheries catch per unit effort

**Figure 7:**
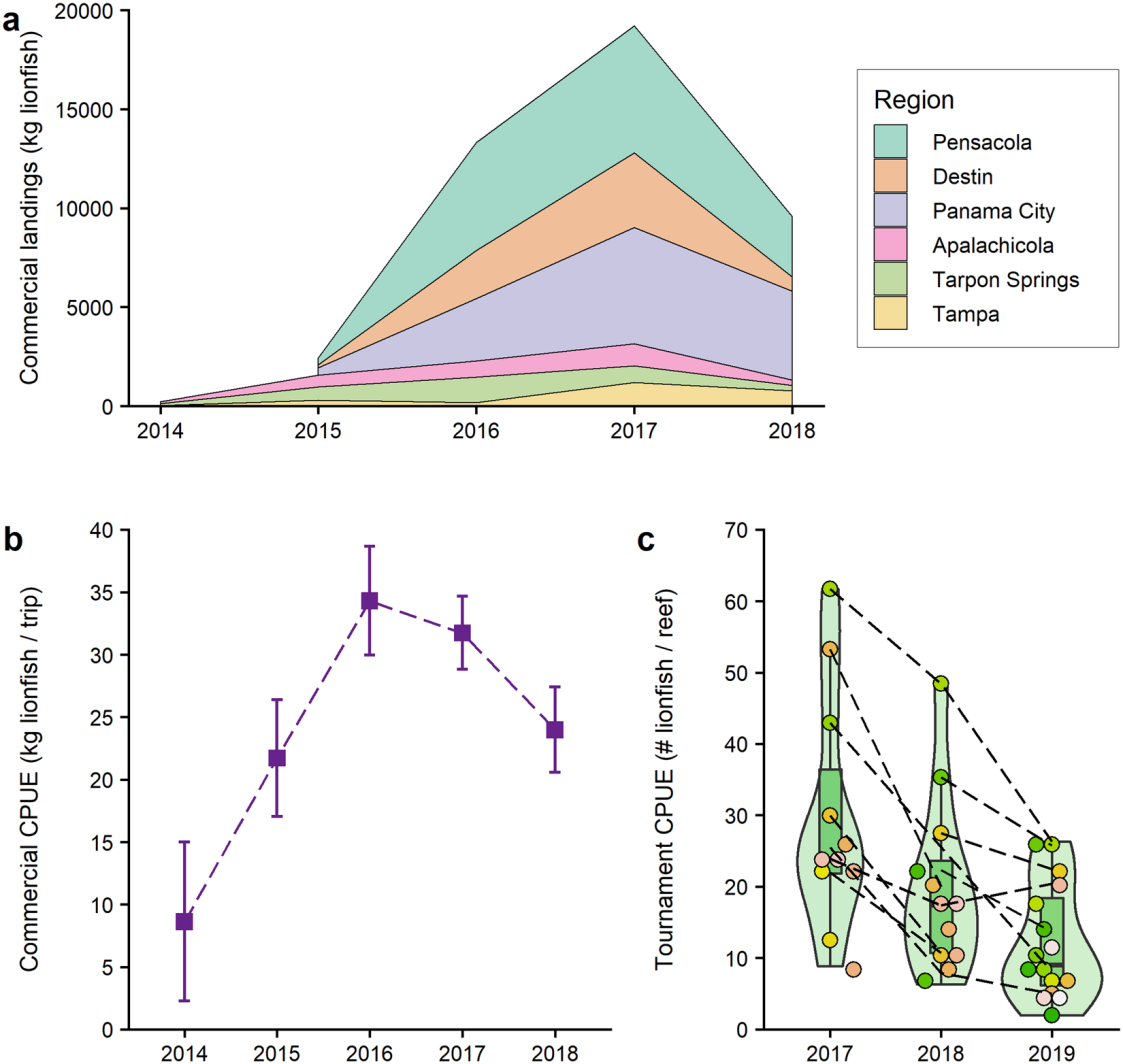
Regional northern Gulf of Mexico (nGOM) lionfish fisheries landings and catch per unit effort (CPUE). (**a**) Total commercial spearfishing landings (kg lionfish) during 2014–2018 by landing port. (**b**) Mean (±95% CI) commercial spearfishing landings CPUE (kg lionfish per commercial trip) during 2014–2018. Data in panels (**a**) and (**b**) were compiled from trip ticket information provided by the Florida Fish and Wildlife Conservation Commission^95^. (**c**) Lionfish tournament CPUE (number of lionfish harvested per reef) from nGOM teams during annual 2-day lionfish spearfishing tournaments conducted May 2017–2019. Points show individual team mean CPUE, dashed lines connect repeated teams, and year-specific violin shape indicates the probability density smoothed with a kernel density estimator.

Regional changes in lionfish populations were examined with fisheries-dependent CPUE information from nGOM commercial spearfishing landings and lionfish tournaments. Commercial lionfish spearfishing CPUE was assessed from Florida Fish and Wildlife Conservation Commission (FWC) trip ticket data. Catches were measured as kg lionfish landed with effort measured per trip. During 2014–2018, a total of 44,731 kg of lionfish were landed in the GOM from 1,529 trips. Over 90% of lionfish were landed in the Florida nGOM ports of Pensacola, Destin, Panama City, and Apalachicola (Fig. 7A). Lionfish commercial landings increased from near-0 in 2014 to almost 20,000 kg in 2017 (Fig. 7A). In 2018, landings then declined by 52% and commercial trips declined by 38% compared to the previous year (Table 3).

**Table 3:** Gulf of Mexico lionfish fisheries catches, effort, and catch per unit effort (CPUE). Differences among years for commercial spearfishing landings CPUE (kg lionfish per commercial trip) and tournament CPUE (number lionfish harvested per reef) were tested with lognormal generalized linear models. Model outputs show the log-linked parameter estimates for mean CPUE by year, 95% confidence intervals (CI) around mean CPUE, t-values (t), and p-values (P). Hypothesis tests (t- and p-values) evaluated changes in mean annual CPUE from the intercept year in each model, i.e., 2015 commercial spearfishing landings CPUE and 2017 tournament CPUE.

CPUE model	Year	Effort: # trips	Total catch (kg lionfish)	Mean CPUE (kg/trip)	95% CI	t	P
Commercial spearfishing landings	2015 *(Intercept)*	112	2432	21.71	15.77–29.89	18.87	**<0.001**
	2016	388	13310	34.30	24.53–47.98	2.66	**0.008**
	2017	605	19204	31.70	22.80–44.29	2.23	**0.026**
	2018	399	9570	23.88	16.72–34.08	0.55	0.582
		**Effort: # reefs**	**Total catch (# lionfish)**	**Mean CPUE (#/reef)**			
Tournament	2017 *(Intercept)*	109	3828	24.87	19.65–31.47	26.73	**<0.001**
	2018	400	8738	13.93	12.19–15.92	−8.71	**<0.001**
	2019	649	9223	9.45	07.96–10.94	−11.62	**<0.001**

Changes in commercial spearfishing landings CPUE were assessed with lognormal GLMs (log linked) to test differences by year (Table 3). GLM results indicate mean commercial spearfishing CPUE increased significantly from 21 kg per trip in 2015 to 31–34 kg per trip in 2016–2017 (Fig. 7B, P < 0.008). In 2018, mean CPUE declined 48% compared to highs observed in 2016. CPUE in 2018 was not significantly different to 2015 despite *c*. 4X higher landings and effort in 2018 (Table 3, P = 0.582).

Lionfish tournament CPUE was evaluated with 21,789 lionfish speared from 1,159 nGOM ARs and NRs during 2017–2019 (Table 3). Catches were measured as the number of lionfish and effort measured as number of reef sites spearfished. Repeated measures from returning teams were incorporated in the tournament mixed model as a random effect and included six teams from 2017 participating in 2018, four teams in 2018 participating in 2019, and two teams that participated in all three years. Lionfish tournament catches and effort increased each year from 2017–2019. In contrast, mean tournament CPUE peaked in 2017 then declined through 2019 (Table 3). GLMM results indicate CPUE declined by 44% in 2018 compared to 2017 (P < 0.001) with all six repeating teams having lower CPUE (Fig. 7C). Mean tournament CPUE declined an additional 18% in 2019 (P < 0.001) with six of seven repeating teams having lower CPUE.

## Discussion

Rapid and substantial changes in invasive lionfish populations were observed in 2018 over a broad area of the nGOM continental shelf. Population densities declined >77% on initially high-density ARs in our study areas, which previously had the highest reported lionfish densities within the invaded range^19,51^. Similar levels of decline were observed on NRs, which had two-orders of magnitude lower lionfish densities but make up >99% of the region’s reef habitat^53^. Concurrently, an approximate 80% drop in the proportion of age-0 lionfish occurred in early summer 2018, which would be the expected period of high recruitment for subtropical lionfish^54^. These population and recruitment reductions likely drove regional declines observed in total lionfish commercial spearfishing landings (52% decline), commercial spearfishing CPUE (48% decline), and tournament CPUE (62% decline). Collectively, these changes indicate total mortality in the region increased by orders of magnitude compared to prior years^27,55,56^ and that nGOM lionfish populations may have experienced a population crash^4,5^.

Population declines and fluctuations are common in invasive species^1^, but their causes are often not understood^4^. In nGOM lionfish, the rapid emergence of the ulcerative skin disease about 1 y prior to the population declines reported here appears to implicate the disease as a causative mechanism. Given that disease etiology and its mortality rates remain unknown^50^, the disease-driver hypothesis for the declines is based on correlative evidence. It is apparent that population declines were density-dependent, suggesting disease transmission was also density-dependent. The first reports of diseased lionfish were from nGOM high-density ARs^49^ and these had by far the greatest magnitude of population declines. By the end of 2018, lionfish density on initially high-density ARs was not significantly different than lionfish density on initially low-density ARs that had undergone prior lionfish removals. Moreover, disease prevalence was as high as 40% in late summer 2017 and the greatest declines in relative condition were observed in ulcerated female lionfish compared to non-ulcerated females. This high disease prevalence coincided with expected seasonal highs in nGOM lionfish reproductive output when waters are warmest. Northern GOM lionfish gonadosomatic index (i.e., proportion of gonad weight to fish weight) peaks in August at *c*. 6%^17^, suggesting reallocation of energetic resources from egg production to combat infection may have decreased fitness and egg production^57^. The rebound in the proportion of age-0 fish observed in fall 2018 to levels similar to fall 2014 may then be explained by the subsided disease prevalence observed earlier that year.

Other factors also may have caused or contributed to the lionfish population declines, including control by native predators, fisheries removals, and/or severe weather disturbances. Although quantifying indirect effects of competition by native predators on lionfish is challenging^20^, there is evidence that they compete with lionfish for space^58^ and prey^27^. Invasive lionfish have also been occasionally reported in stomachs of native Caribbean predators^59,60^ and grouper biomass has been shown to be inversely correlate to lionfish biomass^61^. However, subsequent studies with larger and more comprehensive sampling indicated no correlation between lionfish and native predators^32,34^. Furthermore, lionfish have not been documented in stomach samples of nGOM predators^27^. The current general consensus is that lionfish venomous spines deter predators^20,21^ and inter- and intraspecific (i.e., cannibalism) predation rates are too low to control lionfish populations^20,31,33^. Thus, fishery removals are widely encouraged as the primary means for controlling lionfish densities^20,21^. High removal effort can control lionfish densities on local reefs^22,62,63^, but population models have indicated regional nGOM fishing intensity has been well below the levels estimated to cause recruitment overfishing^27,55,64^. While we observed nGOM commercial landings increased *c*. 8X between 2015 to 2017, it was estimated that an approximate 100X increase in fishery removals from 2015 levels would be necessary to achieve a fishing mortality rate that would substantially reduce regional lionfish biomass^27^. Moreover, the strong declines on high-density reefs indicate population effects from removals may be mitigated by density-dependent population compensation. Analogous compensatory effects were also observed in The Bahamas after the passage of a major hurricane resulted in substantial increases in lionfish density on reefs treated with the highest removal effort prior to the storm^65^.

Strong tropical weather systems may have affected lionfish larval transport, caused mortality, or redistributed lionfish (e.g., from high-density to low density reefs). The 2017–2018 tropical weather seasons included the passage of three hurricanes in the nGOM in 2017 (Harvey, Irma, and Nate) and, in 2018, Hurricane Michael was the strongest (category 5) storm on record to make landfall in northwest Florida. Previously, hurricanes have appeared to help lionfish spread and establish lionfish in the Western Atlantic. Larval transport to southeast Florida and The Bahamas was accelerated by hurricanes in the 2000s^66^ and lionfish density on reefs monitored in The Bahamas increased 4–7X following the passage of a category 3 hurricane Irene^65^. However, biophysical models show lionfish larval transport in the nGOM differs from the Caribbean^67^. Relatively weak currents in the northeastern GOM, and its proximity to the GOM Loop Current, indicate the region is likely both a lionfish source and sink^68^. These oceanographic dynamics, which likely facilitated a rapid nGOM invasion and record high densities observed during 2010–2015^67,68^, may have been disrupted by hurricanes during 2017 and/or 2018. Notably, the passage of category 3 hurricane Irma through the nGOM in September 2017, about 1 month after the first record of the ulcerative skin disease, resulted in a cold-water upwelling that decreased bottom temperatures by >11°C (monitoring location 30.14°N, 86.60°W, K. Dahl, Univ. of Florida, unpubl. data). While the minimum bottom temperature (*c*. 16°C) remained within the tolerance range of lionfish^13^, we do not know the potential interaction effects from thermal stress, the disease, and other potential stressors.

If the disease caused the observed lionfish population declines, it would indicate a potential end of their release from parasitic or pathogenic controls in the western Atlantic. Results from multiple studies conducted earlier in the invasion indicated lionfish had low susceptibility to native-range harmful micro- and macro- parasites^35–39^, possibly because lionfish-associated bacterial communities are considerably different from that of native species^69^. Furthermore, these communities exhibit disease resistance to known fish pathogens^70^. Such release from native-range infectious organisms is common for established non-native species^71^ and often contributes to invasion success for introduced species^72^. However, the colonization time theory predicts invaders will acquire new parasites or pathogens within their invaded range as time since introduction increases^71,73^. For example, European round goby *Neogobius menalostomus* in the Great Lakes had considerably lower parasite loads 1 y post establishment as compared to either native *N. menalostomus* populations or indigenous Great Lakes gobies, but had comparable parasite loads 15 y later^74^. At this time, the role of enemy release (i.e., from predators, parasites, and pathogens) followed by enemy accumulation as a determinant of invasive species success and population dynamics is under active research and debate^5^.

More time will be necessary to understand how native communities will be affected by changes in the lionfish populations or the lionfish epizootic. Recent experimental lionfish removal studies have indicated declines in lionfish density result in a mixture of positive^62,75^, negative^65^, and undetectable^22,63^ effects on native prey and competitor fish biomass. Possible explanations for the lack of expected, positive results include the following: recruitment and succession dynamics operate on longer time scales than could be observed in the study period; ecological damages persist after lionfish are removed^4,76^; the density of undetected lionfish in the system is greater than the critical density to achieve community-level benefits^40,51^; or, ecological benefits are confounded by natural^65^ or anthropogenic^22^ disturbances. Furthermore, population fluctuations caused by intermittent epizootic outbreaks, severe storms, or other unforeseen causes will likely have unpredictable higher-order effects in marine consumer-resource interactions. Food web modeling of the nGOM ecosystem indicates complex trophic dynamics exist between native species and lionfish^27^ due to lionfish occupying a mid-level trophic position and having a generalist and adaptable diet^11,22^. Future research on lionfish population dynamics, carrying capacity, and the effectiveness of mitigation efforts will be required to understand the interactions of lionfish removals and natural controls in achieving target lionfish densities, with the ultimate goal of benefiting native species. There is also a potential of pathogen spillback (i.e., when non-indigenous species act as new hosts for native infectious agents to cause new infections in native fauna) of the lionfish ulcerative disease to native species^77^. While the FWC Fish Kill hotline has received reports of ulcerated native fishes with scale loss and visible muscle (pers. comm., Florida Fish and Wildlife Research Institute), these records predate the lionfish skin disease and a low prevalence of ulcers on native fishes is expected from diseases already present^78^. An increase in skin ulcer prevalence was also observed in some nGOM species following the 2010 Deepwater Horizon oil spill^79^. Given the undetermined etiology for the lionfish ulcerative skin disease, the potential for spillback to native fishes is currently unknown^49,50^.

Long-term scientific monitoring will continue to be critical in our understanding of lionfish and invasive species population dynamics. Invasive species boom-bust cycles can be solitary with permanent effects (e.g., St. Matthew Island reindeer *Rangifer tarandus*^76^) or recurrent^5^ (e.g., Hudson river zebra mussels *Dreissena polymorpha*^80^). The history of Australia’s invasive rabbits *Oryctolagus cuniculus* provides a well-documented example of invasive species populations driven by epizootic control. Intentional introductions of the myxoma virus in the 1950s^81^ and rabbit hemorrhagic disease virus in 1995^82^ each decreased rabbit densities >90%. Each population decline was then followed by host–pathogen coevolution^83^ and rabbit population recoveries^84^. Today, naturally recurring outbreaks of both diseases persist with considerable variation in geography, season, and mortality^85^. For nGOM lionfish, it is unclear if populations will increase to previous levels and thus complete a boom-bust cycle. We postulate that a solitary population crash is unlikely given their regional spread^18–20^ and ability for non-local replenishment^67,86^. By fall 2018, lionfish size composition and the proportion of new recruits returned to similar levels observed in 2014, prior to the population declines. This recruitment signal could indicate the initial signs of a population rebound. A lag period would be expected^4^ as new recruits grow and mature, which could explain the continued decline in 2019 tournament CPUE. A lionfish population recovery would be facilitated by the same physiological attributes that contributed to their invasion success^11–14^ and opportunistic life history characteristics that facilitate rapid population growth, including early sexual maturity^16,17^ and high reproductive output^16,17^. That said, invasive species outcomes are notoriously difficult to forecast^4^. A lionfish population recovery could be prevented by high fishing pressure^62,75,87^ or if reef systems are now less hospitable to lionfish after their initial invasion depleted prey species^23,24,42^, restructured reef communities^22–24^, or altered ecosystem processes^25–27^.

## Methods

### Documenting ulcer prevalence on sampled nGOM lionfish

Following the first documented report of the ulcerative skin disease on August 5, 2017, gross examination for skin ulcers was conducted for all sampled lionfish to estimate the proportion of visually identified diseased individuals present within a given sample. Ulcers were macroscopically identified by examining both sides of a given lionfish for characteristic scale loss, necrotic tissue, or muscle visible through holes in the skin (Fig. 2). No partial ulcer development was observed; however, ulcer staging was not attempted. The proportion of ulcerated lionfish was estimated opportunistically from nGOM commercial spearfishing landings during August, October, and December 2017. No other data (e.g., length, sex) were collected from these samples. From January 2018 to June 2019, the proportion of ulcerated lionfish was estimated during monthly size composition sampling, described below.

### Relative condition of ulcerated lionfish compared to apparently healthy and previously collected specimens

Lionfish (n = 338) were sampled by researchers via spearfishing in October and November 2017 from nGOM NRs approximately 20 km south of Destin, FL, USA (Fig. 1). Lionfish collection followed humane sampling protocol with euthanasia via pithing the brain case, as reviewed and approved by the University of Florida’s Institutional Animal Care and Use Committee (UF IACUC Protocol #201810225). Lionfish were macroscopically examined for evidence of skin ulcers, measured to nearest mm total length (TL), weighed to the nearest 0.1 g, and sexed by visual identification of internal gonads.

Predicted weight-at-length ($\hat{{\rm{W}}}$) was computed for each lionfish with a non-linear length-weight model (Eq. 1)

1 $$\hat{{\rm{W}}}i=aT{L}^{b}$$

Allometric parameters *a* and *b* were estimated by fitting a log-linear length-weight equation (Eq. 2) with 598 lionfish sampled prior to the appearance of the lionfish disease. These lionfish were sampled during 2013–2016 from similar nGOM NRs (Fig. 1) and with the same methods described above^19^.

2 $$\mathrm{Log}\,{\hat{{\rm{W}}}}_{i}=\,\log (a)+b\ast \log (TL)$$

Differences between the $\log \,{\hat{{\rm{W}}}}_{i}$ models for males and females were tested with a two-factor ANOVA (Eq. 3)^88^.

3 $$\begin{array}{c}\mathrm{Log}\,{\hat{{\rm{W}}}}_{i} \sim \,{\rm{Normal}}({\rm{\mu }})\\ {\rm{\mu }}=\,\log (TL)+Sex+Sex:\,\log (TL)\end{array}$$

ANOVA results found a significant interaction between log TL and sex (F_(1, 594)_ = 9.14; P = 0.003), indicating a biologically significant difference between the male and female allometric scaling *b* values. Therefore, sex-specific ${\hat{{\rm{W}}}}_{i}$ models were computed. Female ${\hat{{\rm{W}}}}_{i}$ was calculated with *a* = 4.67e-10 and *b* = 3.62; male ${\hat{{\rm{W}}}}_{i}$ was calculated with *a* = 1.66e-10 and *b* = 3.42.

Sex-specific relative condition factor (K_n_) was then estimated for each lionfish sampled in 2017 as the ratio of an individual’s measured weight (*W*_*i*_) to its sex-specific predicted weight-at-length, ${\hat{{\rm{W}}}}_{i}$ (Eq. 4)^89^, as is recommended for within a sample when comparing relative weights for fishes^90^.

4 $${K}_{n}=\frac{{W}_{i}}{{\hat{{\rm{W}}}}_{i}}\,$$

Quantile-quantile (QQ) plots were examined to determine if errors were best fit with a normal, lognormal, Poisson, or negative binomial distribution. QQ plots indicated K_n_ had a normal error distribution, thus differences in mean K_n_ were tested with a two-factor ANOVA. Factors included ulcer presence (ulcerated or non-ulcerated), sex (male or female), and the interaction between ulcer presence and sex (Eq. 5). Group means were then tested with a Tukey HSD test. Analyses were conducted in R (version 3.5.1) with the base stats package. Data manipulation and visualization here and below were performed with R tidyverse packages DPLYR and GGPLOT2. See supplemental material for R code, raw data, and diagnostic plots.

5 $$\begin{array}{c}{K}_{n} \sim \,{\rm{Normal}}({\rm{\mu }})\\ {\rm{\mu }}=Ulcer\,presence+Sex+Ulcer\,presence:Sex\end{array}$$

### Lionfish size composition, recruitment, and disease prevalence

Size composition was examined for nGOM lionfish sampled during 2014–2018. Lionfish were sampled from ARs and NRs offshore of Pensacola, FL; Destin, FL; and Panama City, FL. Sampling was conducted during May–October 2014, May 2015–2017, and May–October 2018, which coincides with the seasonal high recruitment periods for subtropical lionfish^54^. Lionfish (n = 31,073) were sampled via spearfishing during lionfish tournaments and during non-tournament periods (n = 201 sampling events). Harvesters were instructed to spear all lionfish observed on a given reef regardless of size, which was incentivized via prizes for total number of lionfish harvested. Lionfish were measured to the nearest mm TL. Harvesters were informed and consented to information about their catch and effort being used for research purposes. The number of sampling events per month ranged from 2 to 20, the number of lionfish collected per sampling event ranged from 31 to 2,402, and the number of lionfish collected per month ranged from 502 to 8,500.

Length-frequency data were visually examined by month via density plots. Changes in size composition were quantitively evaluated with a logistic regression to evaluate changes in the proportion ($\hat{{\rm{P}}}$) of age-0 lionfish (*N*_*age-0*_) among the population sampled (*N*_*t*_) per sampling event *t*, (i.e., collection day). Using sampling event as the experimental unit prevented overly sensitive significance findings due to large sample size and is recommended for use in length-frequency fisheries data^91^. The length cutoff for newly recruited age-0 lionfish was determined as 172 mm TL based on the mean 95^th^ percentile values for length at age-0 from sagittal otolith aging of nGOM lionfish [i.e., mean between 171 mm based on Dahl *et al*. (2019) and 173 mm based on from Fogg *et al*. (2019)]. Sensitivity analysis indicated that changes in cutoff values between 120 and 178 mm for length at age-0 lionfish did not alter significance of 2018 $\hat{{\rm{P}}}$. Sex was not evaluated as lionfish <180 mm are generally not sexually identifiable via visual examination of gonad^19^.

Differences in $\hat{{\rm{P}}}$ were tested with a binomial GLM (logit link) (Eq. 6). Factors included month (categorical with 6 levels, May–October) nested within year (categorical with 2 levels, 2014 and 2018) and weighted by number of lionfish measured per sampling event with a log link [i.e., log (*N*_*t*_)]. Nesting allowed hypothesis testing 1) between months within the same year and 2) between years within the same month. Models were fit using Laplace approximation and maximum likelihood^92^. Analyses were conducted in R with the LME4 and MASS packages. See supplemental material for code, raw data, and diagnostic plots.

6 $$\begin{array}{c}\hat{{\rm{p}}}=\frac{{N}_{age-0}}{{N}_{t}} \sim {\rm{Binomial}}({\rm{\mu }})\\ {\rm{logit}}({\rm{\mu }})=Month/Year\end{array}$$

### Lionfish population density monitoring with ROV surveys

Lionfish population density during 2016–2018 was estimated from 8 surveys at Okaloosa ARs, 5 surveys at Escambia ARs, and 5 surveys at nGOM NRs. Okaloosa ARs are located *c*. 30 km south of Destin, FL, USA at 29–41 m depths and Escambia ARs are located 32 km south of Pensacola, FL, USA at 25–38 m depths (Fig. 1). Okaloosa and Escambia ARs are part of respective Large Area Artificial Reef Sites (LAARS) deployed by the state of Florida in 2003. They are designated refuge ARs with their coordinates not reported to the public. Reef sites consisted of prefabricated AR modules of three designs: concrete and metal pyramids (3.0 m x 3.0 m, height x base), paired concrete reef balls (1.8 m x 1.8 m), or paired concrete tetrahedrons (1.8 m x 1.8 m). ARs in each area included low-density (<15 lionfish per 100 m2) ARs at which lionfish removal experiments were conducted at 8 Okaloosa sites during 2016^51^ and 9 Escambia sites during 2014^22^. High-density (>25 lionfish per 100 m^2^) ARs were control sites during the previous removal experiments and never had lionfish removed. NRs consisted of relict carbonate structures and included limestone ledges and plateaus, rocks, and gravel in 20–70 m of water and distributed over an approximately 15,000 km^2^ area of the nGOM shelf (Fig. 1). No lionfish removals were conducted at NR sites.

Lionfish density surveys were conducted with a VideoRay Pro4 mini ROV equipped with a 570-line color camera with wide-angle (116°) lens and a GoPro Hero5 high-definition digital camera mounted to the front of the ROV. The ROV was controlled with an integrated control box via the ROV’s tether and real-time ROV movement was observed on a high-resolution monitor with a live feed from the ROV’s video camera. Lighting was provided by twin 20-watt high efficiency halogen lights mounted on the ROV. Digital video of ROV surveys was recorded then read in the laboratory to produce fish counts. ARs were surveyed with a point-count method that utilizes a 15-m wide cylinder with the reef at the center (survey area = 177 m^2^) with fish counts made on opposite sides of the reef, above the reef, then inside of the reef^93^. NRs were surveyed with four orthogonal 25-m long transects flown from a common central point^94^. Transect width was calculated based on ROV height off the bottom (*c*. 1 m) and the 45° angle of the camera relative and used to compute survey area.

GLMMs were computed to test changes in lionfish density over time on Okaloosa ARs, Escambia ARs, and nGOM NRs. Lionfish counts (positive integers) were offset in the model by survey area (m^2^) $\times$ 100 to calculate lionfish density per 100 m^2^. Fixed effects included sample date (treated categorically) nested within treatment group (categorical with two levels for AR GLMMs: low- or high-density) (Eq. 7).

7 $$\begin{array}{rrl}Densit{y}_{OkaloosaARs} & \sim  & Negative\,binomial(\mu )\\ Densit{y}_{EscambiaARs} & \sim  & {\rm{N}}{\rm{e}}{\rm{g}}{\rm{a}}{\rm{t}}{\rm{i}}{\rm{v}}{\rm{e}}\,{\rm{b}}{\rm{i}}{\rm{n}}{\rm{o}}{\rm{m}}{\rm{i}}{\rm{a}}{\rm{l}}(\mu )\\ Densit{y}_{nGOMNRs} & \sim  & {\rm{P}}{\rm{o}}{\rm{i}}{\rm{s}}{\rm{s}}{\rm{o}}{\rm{n}}(\mu )\\ Site & \sim  & N(0,{\sigma }^{2})\\ \log (\mu ) & = & Sample\,Date/Treatment+(1|Site)\end{array}$$

QQ plots indicated error for Okaloosa and Escambia GLMMs was best fit with negative binomial error distributions, which is typical for over-dispersed count data^92^. Error for the NR GLMM was best fit with a Poisson error distribution. To incorporate dependency of observations for repeated measurements of the same reef, individual reef was included as a random effect (random intercept) and assumed to be normally distributed with a mean of 0 and variance σ^2^. Laplace approximation was used to estimate likelihood and information criteria based on GLMM fitting and inference protocols^92^. Models were built in R (version 3.5.1) and with the LME4 and MASS packages. See supplemental material for R code, raw data, and QQ plots.

### Lionfish fisheries catches and CPUE from commercial spearfishing landings and tournaments

Commercial lionfish spearfishing landings data were provided by trip ticket records managed by the FWC for Florida waters^95^. Trips were subset for lionfish food harvest (as opposed to lionfish collected for the aquarium trade) by spearfishing in GOM waters and excluded the Florida Keys. Commercial spearfishing CPUE was computed as kg lionfish landed (44,731 total) per commercial harvest trip (1,529 total). QQ plots indicated commercial CPUE error had a lognormal distribution. Differences in commercial CPUE by year during 2015–2018 (treated categorically) were tested with a log-linked GLM (Eq. 8). The first year of reported commercial harvest in 2014 was excluded due to the low sample size (n = 25) and high variance in catches. Models were fit using Laplace approximation and maximum likelihood^92^ in R (version 3.5.1) with the LME4 and MASS packages. See supplemental material for code, raw data, and QQ plots.

8 $$\begin{array}{c}CPU{E}_{Commercial} \sim {\rm{Lognormal}}({\rm{\mu }})\\ \log ({\rm{\mu }})=\,Year\end{array}$$

Lionfish tournament catches and effort were recorded from annual 2-day lionfish spearfishing tournaments conducted May 2017–2019 from teams that participated in nGOM waters. Tournament divers were informed and consented to information about their catch and spearfishing effort being used for research purposes. Catches (number of lionfish) and effort (number of reef sites) were recorded from 11 teams (44 divers) in 2017, 12 teams (48 divers) in 2018, and 16 teams (64 divers) in 2019. Tournament CPUE was computed as the number of lionfish caught (n = 21,789) per reef site (n = 1,159 total). Removal of all lionfish at a reef site was incentivized with tournament prizes for the most, largest, and smallest lionfish. Consistent catchability among teams was assumed based on high removal efficiency on low complexity nGOM reefs^51^. QQ plots indicated tournament CPUE had lognormal error, similar to commercial spearfishing CPUE. Changes in tournament CPUE during 2017–2019 (treated categorically) were tested with a log-linked GLMM (Eq. 9). Team was included as a random effect (random intercept) given some teams participated in tournaments in multiple years: six teams from 2017 participating in 2018, four teams in 2018 participated in 2019, and two teams that participated in all three years. Models were estimated with Laplace approximation and maximum likelihood^92^ and built in R (version 3.5.1) with the LME4 and MASS packages. See supplemental material for code, raw data, and QQ plots.

9 $$\begin{array}{c}CPU{E}_{Tournament} \sim {\rm{Lognormal}}({\rm{\mu }})\\ Team \sim N(0,{{\rm{\sigma }}}^{2})\\ \log ({\rm{\mu }})=Year+(1|Team)\end{array}$$

## Supplementary information

Supplementary Information.

Supplementary Information1.

Supplementary Information2.

Supplementary Information3.

Supplementary Information4.

Supplementary Information5.

Supplementary Information6.

Supplementary Information7.

Supplementary Information8.

Supplementary Information9.

## Data Availability

Data are publicly available through the Gulf of Mexico Research Initiative Information & Data Cooperative (GRIIDC) at https://data.gulfresearchinitiative.org. All analyses are reproducible with the data and R code provided in the supplementary material.

## References

[1] Garcia-Diaz Pablo (2016). Invasion Ecology, 2nd edition by J. L.Lockwood, M. F.Hoopes and M. P.Marchetti. Wiley-Blackwell, West Sussex, 2013. xi + 444 pp. Price AUD$94.95 (paperback, also available in hardcover and e-book). ISBN 978-1-4443-3364-0. Austral Ecology.

[2] Blackburn, T. M. *et al*. A unified classification of alien species based on the magnitude of their environmental impacts. *PLoS Biol*. **12** (2014). 10.1371/journal.pbio.1001850 PMC4011680 24802715

[3] Kolar CS, Lodge DM (2001). Progress in invasion biology: predicting invaders. Trends Ecol. Evol..

[4] Simberloff D, Gibbons L (2004). Now you see them, now you don’t! – Population crashes of established introduced species. Biol. Invasions.

[5] Strayer DL (2017). Boom-bust dynamics in biological invasions: towards an improved application of the concept. Ecol. Lett..

[6] Nei M, Maruyama T, Chakraborty R (1975). The genetic bottleneck effect and genetic variability in populations. Evolution (N. Y)..

[7] Zhan A (2012). Complex genetic patterns in closely related colonizing invasive species. Ecol. Evol..

[8] Lee KA, Klasing KC (2004). A role for immunology in invasion biology. Trends Ecol. Evol..

[9] Lambrinos JG (2004). How interactions between ecology and evolution influence contemporary invasion dynamics. Ecology.

[10] Lee CE (2002). Evolutionary genetics of invasive species. Trends Ecol. Evol..

[11] Peake J (2018). Feeding ecology of invasive lionfish (*Pterois volitans* and *Pterois miles*) in the temperate and tropical western Atlantic. Biol. Invasions.

[12] Rojas-Vélez S, Tavera J, Acero A (2019). Unraveling lionfish invasion: is *Pterois volitans* truly a morphologically novel predator in the Caribbean?. Biol. Invasions.

[13] Barker BD, Horodysky AZ, Kerstetter DW (2018). Hot or not? Comparative behavioral thermoregulation, critical temperature regimes, and thermal tolerances of the invasive lionfish *Pterois* sp. versus native western North Atlantic reef fishes. Biol. Invasions.

[14] Jud ZR, Nichols PK, Layman CA (2015). Broad salinity tolerance in the invasive lionfish *Pterois* spp. may facilitate estuarine colonization. Environ. Biol. Fishes.

[15] Fogg AQ (2019). Comparison of age and growth parameters of invasive red lionfish (*Pterois volitans*) across the northern Gulf of Mexico. Fish. Bull..

[16] Gardner PG, Frazer TK, Jacoby CA, Yanong RPE (2015). Reproductive biology of invasive lionfish (*Pterois* spp.). Front. Mar. Sci..

[17] Fogg A, Brown-Peterson N, Peterson M (2017). Reproductive life history characteristics of invasive red lionfish (*Pterois volitans*) in the northern Gulf of Mexico. Bull. Mar. Sci..

[18] Green SJ, Côté IM (2009). Record densities of Indo-Pacific lionfish on Bahamian coral reefs. Coral Reefs.

[19] Dahl K, Edwards M, Patterson WF (2019). Density-dependent condition and growth of invasive lionfish in the northern Gulf of Mexico. Mar. Ecol. Prog. Ser..

[20] Côté IM, Smith NS (2018). The lionfish *Pterois* sp. invasion: has the worst-case scenario come to pass?. J. Fish Biol..

[21] Hixon M, Green S, Albins M, Akins J, Morris J (2016). Lionfish: a major marine invasion. Mar. Ecol. Prog. Ser..

[22] Dahl KA, Patterson WF, Snyder RA (2016). Experimental assessment of lionfish removals to mitigate reef fish community shifts on northern Gulf of Mexico artificial reefs. Mar. Ecol. Prog. Ser..

[23] Albins M (2015). Invasive Pacific lionfish *Pterois volitans* reduce abundance and species richness of native Bahamian coral-reef fishes. Mar. Ecol. Prog. Ser..

[24] Green SJ, Akins JL, Maljković A, Côté IM (2012). Invasive lionfish drive Atlantic coral reef fish declines. PLoS One.

[25] Lesser MP, Slattery M (2011). Phase shift to algal dominated communities at mesophotic depths associated with lionfish (*Pterois volitans*) invasion on a Bahamian coral reef. Biol. Invasions.

[26] Kindinger, T. L. & Albins, M. A. Consumptive and non-consumptive effects of an invasive marine predator on native coral-reef herbivores. *Biol. Invasions* **19**, (2017).

[27] Chagaris D (2017). An ecosystem-based approach to evaluating impacts and management of invasive lionfish. Fisheries.

[28] Kulbicki M (2012). Distributions of Indo-Pacific lionfishes *Pterois* spp. in their native ranges: implications for the Atlantic invasion. Mar. Ecol. Prog. Ser..

[29] Darling ES, Green SJ, O’Leary JK, Côté IM (2011). Indo-Pacific lionfish are larger and more abundant on invaded reefs: A comparison of Kenyan and Bahamian lionfish populations. Biol. Invasions.

[30] Benkwitt CE (2017). Is the lionfish invasion waning? Evidence from The Bahamas. Coral Reefs.

[31] Hackerott S (2013). Native predators do not influence invasion success of Pacific lionfish on Caribbean reefs. PLoS One.

[32] Bejarano S, Lohr K, Hamilton S, Manfrino C (2015). Relationships of invasive lionfish with topographic complexity, groupers, and native prey fishes in Little Cayman. Mar. Biol..

[33] Valdivia A, Bruno JF, Cox CE, Hackerott S, Green SJ (2014). Re-examining the relationship between invasive lionfish and native grouper in the Caribbean. PeerJ.

[34] Anton A, Simpson MS, Vu I (2014). Environmental and biotic correlates to lionfish invasion success in Bahamian coral reefs. PLoS One.

[35] Fogg, A. Q., Ruiz, C. F., Curran, S. S. & Bullard, S. A. Parasites from the red lionfish, *Pterois volitans* from the Gulf of Mexico. *Gulf Caribb. Res*. **27**, SC 1-5 (2016).

[36] Sellers AJ, Ruiz GM, Leung B, Torchin ME (2015). Regional variation in parasite species richness and abundance in the introduced range of the invasive lionfish, *Pterois volitans*. PLoS One.

[37] Tuttle LJ, Sikkel PC, Cure K, Hixon MA (2017). Parasite-mediated enemy release and low biotic resistance may facilitate invasion of Atlantic coral reefs by Pacific red lionfish (*Pterois volitans*). Biol. Invasions.

[38] Sikkel PC, Tuttle LJ, Cure K, Coile AM, Hixon MA (2014). Low susceptibility of invasive red lionfish (*Pterois volitans*) to a generalist ectoparasite in both its introduced and native ranges. PLoS One.

[39] Loerch SM, McCammon AM, Sikkel PC (2015). Low susceptibility of invasive Indo-Pacific lionfish *Pterois volitans* to ectoparasitic *Neobenedenia* in the eastern Caribbean. Environ. Biol. Fishes.

[40] Benkwitt CE (2015). Non-linear effects of invasive lionfish density on native coral-reef fish communities. Biol. Invasions.

[41] Benkwitt C (2016). Invasive lionfish increase activity and foraging movements at greater local densities. Mar. Ecol. Prog. Ser..

[42] Ingeman KE (2016). Lionfish cause increased mortality rates and drive local extirpation of native prey. Marine Ecology Progress Series.

[43] Dahl KA (2018). Genotyping confirms significant cannibalism in northern Gulf of Mexico invasive red lionfish, *Pterois volitans*. Biol. Invasions.

[44] Benkwitt CE (2013). Density-dependent growth in invasive lionfish (*Pterois volitans*). PLoS One.

[45] Pérez-Portela R (2018). Genetic homogeneity of the invasive lionfish across the Northwestern Atlantic and the Gulf of Mexico based on single nucleotide polymorphisms. Sci. Rep..

[46] Burford Reiskind M. O., Reed E. M. X., Elias A., Giacomini J. J., McNear A. F., Nieuwsma J., Parker G. A., Roberts R. B., Rossi R. E., Stephenson C. N., Stevens J. L., Williams B. E. (2019). The genomics of invasion: characterization of red lionfish (Pterois volitans) populations from the native and introduced ranges. Biological Invasions.

[47] Johnson J, Bird CE, Johnston MA, Fogg AQ, Hogan JD (2016). Regional genetic structure and genetic founder effects in the invasive lionfish: comparing the Gulf of Mexico, Caribbean and North Atlantic. Mar. Biol..

[48] White TA, Perkins SE (2012). The ecoimmunology of invasive species. Funct. Ecol..

[49] Harris HE (2018). First report of an emerging ulcerative skin disease in invasive lionfish. UF/IFAS Ext. Electron. Data Inf. Source.

[50] Ahasan, M. S. *et al*. Determining the etiology of an emerging ulcerative disease in invasive lionfish. In 8th International Symposium on Aquatic Animal Health, 10.13140/RG.2.2.26050.63680 (2018).

[51] Harris HE, Patterson WF, Ahrens RNM, Allen MS (2019). Detection and removal efficiency of invasive lionfish in the northern Gulf of Mexico. Fish. Res..

[52] Dahl KA, Patterson WF (2014). Habitat-specific density and diet of rapidly expanding invasive red lionfish, *Pterois volitans*, populations in the northern Gulf of Mexico. PLoS One.

[53] Karnauskas M (2017). Red snapper distribution on natural habitats and artificial structures in the northern Gulf of Mexico. Mar. Coast. Fish..

[54] Johnson EG, Swenarton MK (2016). Age, growth and population structure of invasive lionfish (Pterois volitans/miles) in northeast Florida using a length-based, age-structured population model. PeerJ.

[55] Barbour, A. B., Allen, M. S., Frazer, T. K. & Sherman, K. D. Evaluating the potential efficacy of invasive lionfish (*Pterois volitans*) removals. *PLoS One* **6**, (2011). 10.1371/journal.pone.0019666 PMC3091870 21572951

[56] Morris JA, Shertzer KW, Rice JA (2011). A stage-based matrix population model of invasive lionfish with implications for control. Biol. Invasions.

[57] Ramsay JM, Watral V, Schreck CB, Kent ML (2009). *Pseudoloma neurophilia* infections in zebrafish (*Danio rerio*): effects of stress on survival, growth, and reproduction. Dis. Aquat. Organ..

[58] Ellis R, Faletti M (2016). Native grouper indirectly ameliorates the negative effects of invasive lionfish. Mar. Ecol. Prog. Ser..

[59] Diller JL, Frazer TK, Jacoby CA (2014). Coping with the lionfish invasion: evidence that naïve, native predators can learn to help. J. Exp. Mar. Bio. Ecol..

[60] Maljković A, Van Leeuwen TE, Cove SN (2008). Predation on the invasive red lionfish, *Pterois volitans* (Pisces: Scorpaenidae), by native groupers in the Bahamas. Coral Reefs.

[61] Mumby PJ, Harborne AR, Brumbaugh DR (2011). Grouper as a natural biocontrol of invasive lionfish. PLoS One.

[62] Green SJ (2014). Linking removal targets to the ecological effects of invaders: a predictive model and field test. Ecol. Appl..

[63] Harms-Tuohy C, Appeldoorn R, Craig M (2018). The effectiveness of small-scale lionfish removals as a management strategy: effort, impacts and the response of native prey and piscivores. Manag. Biol. Invasions.

[64] Morris JA, Shertzer KW, Rice JA (2010). A stage-based matrix population model of invasive lionfish with implications for control. Biol. Invasions.

[65] Smith NS, Green SJ, Akins JL, Miller S, Côté IM (2017). Density-dependent colonization and natural disturbance limit the effectiveness of invasive lionfish culling efforts. Biol. Invasions.

[66] Johnston MW, Purkis SJ (2015). Hurricanes accelerated the Florida-Bahamas lionfish invasion. Glob. Chang. Biol..

[67] Johnston M, Purkis S (2015). A coordinated and sustained international strategy is required to turn the tide on the Atlantic lionfish invasion. Mar. Ecol. Prog. Ser..

[68] Johnston MW, Bernard AM, Shivji MS (2017). Forecasting lionfish sources and sinks in the Atlantic: are Gulf of Mexico reef fisheries at risk?. Coral Reefs.

[69] Stevens JL, Olson JB (2013). Invasive lionfish harbor a different external bacterial community than native Bahamian fishes. Coral Reefs.

[70] Stevens J, Jackson R, Olson J (2016). Bacteria associated with lionfish (*Pterois volitans/miles* complex) exhibit antibacterial activity against known fish pathogens. Mar. Ecol. Prog. Ser..

[71] Torchin ME, Lafferty KD, Dobson AP, McKenzie VJ, Kuris AM (2003). Introduced species and their missing parasites. Nature.

[72] Torchin ME, Lafferty KD, Kuris AM (2002). Parasites and marine invasions. Parasitology.

[73] Blakeslee AMH, Fowler AE, Keogh CL (2013). Marine invasions and parasite escape: updates and new perspectives. Adv. Mar. Biol..

[74] Gendron AD, Marcogliese DJ, Thomas M (2012). Invasive species are less parasitized than native competitors, but for how long? The case of the round goby in the Great Lakes-St. Lawrence Basin. Biol. Invasions.

[75] Peiffer F, Bejarano S, Palavicini de Witte G, Wild C (2017). Ongoing removals of invasive lionfish in Honduras and their effect on native Caribbean prey fishes. PeerJ.

[76] Klein DR (1968). The introduction, increase, and crash of reindeer on St. Matthew Island. J. Wildl. Manage..

[77] Kelly DW, Paterson RA, Townsend CR, Poulin R, Tompkins DM (2009). Parasite spillback: a neglected concept in invasion ecology?. Ecology.

[78] Ziskowski JJ (1987). Disease in commercially valuable fish stocks in the Northwest Atlantic. Mar. Pollut. Bull..

[79] Murawski SA, Hogarth WT, Peebles EB, Barbeiri L (2014). Prevalence of external skin lesions and polycyclic aromatic hydrocarbon concentrations in Gulf of Mexico fishes, post-Deepwater. Horizon. Trans. Am. Fish. Soc..

[80] Pace, M. L., Strayer, D. L., Fischer, D. & Malcom, H. M. Recovery of native zooplankton associated with increased mortality of an invasive mussel. *Ecosphere* **1** (2010).

[81] Ratcliffe FN, Myers K, Fennessy BV, Calaby JH (1952). Myxomatosis in Australia: a step towards the biological control of the rabbit. Nature.

[82] Mutze G, Cooke B, Alexander P (1998). The initial impact of rabbit hemorrhagic disease on European rabbit populations in South Australia. J. Wildl. Dis..

[83] Kerr PJ (2012). Myxomatosis in Australia and Europe: a model for emerging infectious diseases. Antiviral Res..

[84] Mutze G (2014). Recovery of South Australian rabbit populations from the impact of rabbit haemorrhagic disease. Wildl. Res..

[85] Mutze Greg, Bird Peter, Cooke Brian, Henzell Robert (2008). Geographic and Seasonal Variation in the Impact of Rabbit Haemorrhagic Disease on European Rabbits, Oryctolagus cuniculus, and Rabbit Damage in Australia. Lagomorph Biology.

[86] Kitchens LL (2017). Occurrence of invasive lionfish (*Pterois volitans*) larvae in the northern Gulf of Mexico: characterization of dispersal pathways and spawning areas. Biol. Invasions.

[87] Frazer TK, Jacoby CA, Edwards MA, Barry SC, Manfrino CM (2012). Coping with the lionfish invasion: can targeted removals yield beneficial effects?. Rev. Fish. Sci..

[88] Ogle Derek H. (2018). Weight-Length Relationships. Introductory Fisheries Analyses with R.

[89] Le Cren ED (1951). The length-weight relationship and seasonal cycle in gonad weight and condition in the Perch (*Perca fluviatilis*). J. Anim. Ecol..

[90] Froese R (2006). Cube law, condition factor and weight-length relationships: history, meta-analysis and recommendations. J. Appl. Ichthyol..

[91] Neumann, R. M. & Allen, M. S. Size Structure. In *Analysis and Interpretation of Freshwater Fisheries Data* (eds. Guy, C. S. & Brown, M. L.) (American Fisheries Society, 2007).

[92] Bolker BM (2009). Generalized linear mixed models: a practical guide for ecology and evolution. Trends Ecol. Evol..

[93] Patterson, W. F. III., Dance, M. A. & Addis, D. T. Development of a remotely operated vehicle based methodology to estimate fish community structure at artificial reef sites in the northern Gulf of Mexico. *Proc. 61st Gulf Caibb. Fish. Inst*. 263–270 (2009).

[94] Patterson, W. F. III., Tarnecki, J. H., Addis, D. T. & Barbieri, L. R. Reef fish community structure at natural versus artificial reefs in the northern Gulf of Mexico. *Proc. 66th Gulf Caribb. Fish. Inst*. 4–8 (2013).

[95] Florida Fish and Wildlife Conservation Commission. 2010–2018 commercial fishery landings data through batch 1402, https://myfwc.com/research/saltwater/fishstats/commercial-fisheries/landings-in-florida/ (2019).

